# Machine learning-based identification of concomitant stroke and prognostic analysis in patients with Guillain-Barré syndrome: a retrospective study

**DOI:** 10.3389/fimmu.2026.1790415

**Published:** 2026-05-07

**Authors:** Yue Zhou, Yutong Wu, Shuxin Wang, Cheng Ye, Lingxu Xu, Xiao Zhao, Dongsheng Ye, Siyu Li, Li Xiao, Zhaoyou Meng

**Affiliations:** 1 Department of Neurology, Second Affiliated Hospital of Army Medical University, Chongqing, China; 2 Department of Neurology, First Affiliated Hospital of Army Medical University, Chongqing, China

**Keywords:** ANN, Guillain-Barré syndrome, machine learning, prediction model, stroke

## Abstract

**Background:**

Guillain-Barré syndrome (GBS) constitutes an immune-mediated inflammatory polyradiculoneuropathy. Stroke may coexist with GBS during the same clinical episode, but the associated clinical predictors remain insufficiently characterized. The present investigation therefore sought to construct a machine learning-based model for identifying concomitant stroke in patients with GBS.

**Methods:**

This retrospective cohort study included 260 patients with GBS who received care at the Second Affiliated Hospital of Army Medical University from January 1, 2015, to December 31, 2024. All candidate predictors were collected at admission. Feature selection was conducted using LASSO regression, and seven machine learning algorithms were developed and compared. An independent external validation cohort of 60 patients was obtained from the First Affiliated Hospital during the same period. Patients were subsequently grouped according to model-estimated probabilities, and short-term functional outcomes were compared between groups.

**Results:**

Nine clinical predictors were selected to construct seven machine learning models. The neural network architecture exhibited the best performance for identifying concomitant stroke. Internal validation yielded an AUROC of 0.838 (95% CI: 0.739–0.923) for the optimal model. Sensitivity analysis excluding patients with documented prior stroke showed comparable performance. For all outcome measures, time displayed a substantial primary impact (p < 0.001), while interactive terms stayed statistically non-significant (p > 0.05).

**Conclusion:**

The ANN model showed good performance for admission-time identification of concomitant stroke in patients with GBS and distinguished clinically different functional profiles among model-defined groups. Notably, while the longitudinal interaction between temporal factors and assigned risk strata did not achieve statistical significance, the stratification methodology successfully discerned clinically distinct outcome profiles in GBS.

## 1 Introduction

Guillain-Barré syndrome (GBS) constitutes an immune-mediated inflammatory polyradiculoneuropathy, manifesting principally as acute symmetric flaccid limb paralysis (1). Worldwide incidence approximates 100,000 novel cases annually (2), China’s rate specifically registers about 0.67 per 100,000 person-years (3). Data from the International Guillain-Barré Syndrome Outcome Study (IGOS) reveal that during the disease’s most severe phase, 19% of affected individuals necessitate support with mechanical ventilation, and 76% lose independent ambulation capacity (4). Although prognosis remains generally favorable, mortality or severe residual disability affects a notable proportion.

Stroke represents an acute disruption in cerebral blood circulation, typically caused by vascular occlusion or hemorrhage, leading to localized ischemic injury or hypoxic damage within brain tissues. As a major contributor to worldwide mortality and persistent disability, this condition poses a substantial global health burden (5). Common clinical presentations include unilateral motor or sensory deficits such as limb weakness or numbness. Patients may also experience visual disturbances—ranging from monocular or binocular vision loss to blurred eyesight—as well as impaired balance and coordination (6). Well-documented risk factors for cerebrovascular disease, such as hypertension, diabetes, dyslipidemia, and advanced age, constitute fundamental elements in the pathogenesis of stroke. A growing body of clinical and experimental research continues to highlight the involvement of systemic inflammatory responses and altered immune regulation in the onset and advancement of stroke pathology (7).

Acute presentations of GBS and stroke frequently converge—flaccid limb paresis, dysphagia, and slurred speech are common to both—thereby complicating early bedside discrimination (8). This symptomatic similarity often results in GBS being misdiagnosed as stroke at initial clinical presentation, potentially delaying critical immunomodulatory interventions and extending the duration of neurological deterioration, ultimately adversely affecting patient prognosis (8). Reported cases indicate that GBS can develop following central nervous system injuries such as intracerebral hemorrhage (9), and stroke may also be identified in patients with GBS during the same clinical episode (10). However, in retrospective cohorts, the temporal sequence between these two conditions is often difficult to determine with certainty, particularly when prior neuroimaging is unavailable. This reciprocal clinical relationship implies a complex underlying immuno-inflammatory mechanism. From a pathophysiological perspective, systemic immune activation and inflammatory responses may contribute to a shared biological milieu linking peripheral neuropathy and cerebrovascular injury (1, 11, 12). Therefore, developing a robust predictive framework for the identification and risk stratification of concomitant stroke in the GBS population represents an essential research priority.

Machine-learning (ML) prognostic frameworks provide three principal strengths for biomedical inquiry. Firstly, they autonomously uncover intricate, non-linear relationships and variable interactions embedded in high-dimensional clinical datasets without requiring manually specified rules. Secondly, by integrating regularization and cross-validation, these models achieve strong generalizability, ensuring that patterns learned from training data remain valid when applied to previously unseen patient populations. Thirdly, ML algorithms unite computational speed with automated feature-optimization capacity, enabling seamless fusion of multi-source, heterogeneous healthcare information. Together, these attributes furnish robust, data-driven support for early risk stratification and precision intervention in clinical practice (13, 14).

This multicenter retrospective study utilized patient data from two tertiary hospitals to develop a ML-based predictive model for concomitant stroke in individuals with GBS and to identify pivotal risk factors. Based on the model-derived risk probabilities, patients were categorized into high-risk and low-risk subgroups. This stratification enabled the assessment of the impact of coexisting stroke on patient prognosis, specifically examining whether the coexistence of both conditions was associated with poorer neurological recovery. Furthermore, the study retrospectively evaluated the short-term efficacy of different immunomodulatory therapies—such as IVIg and PE—across these distinct risk strata. The findings aim to provide evidence to support the development of individualized treatment strategies for patients with this complex comorbidity.

## 2 Methods

### 2.1 Data source

**Figure 1:**
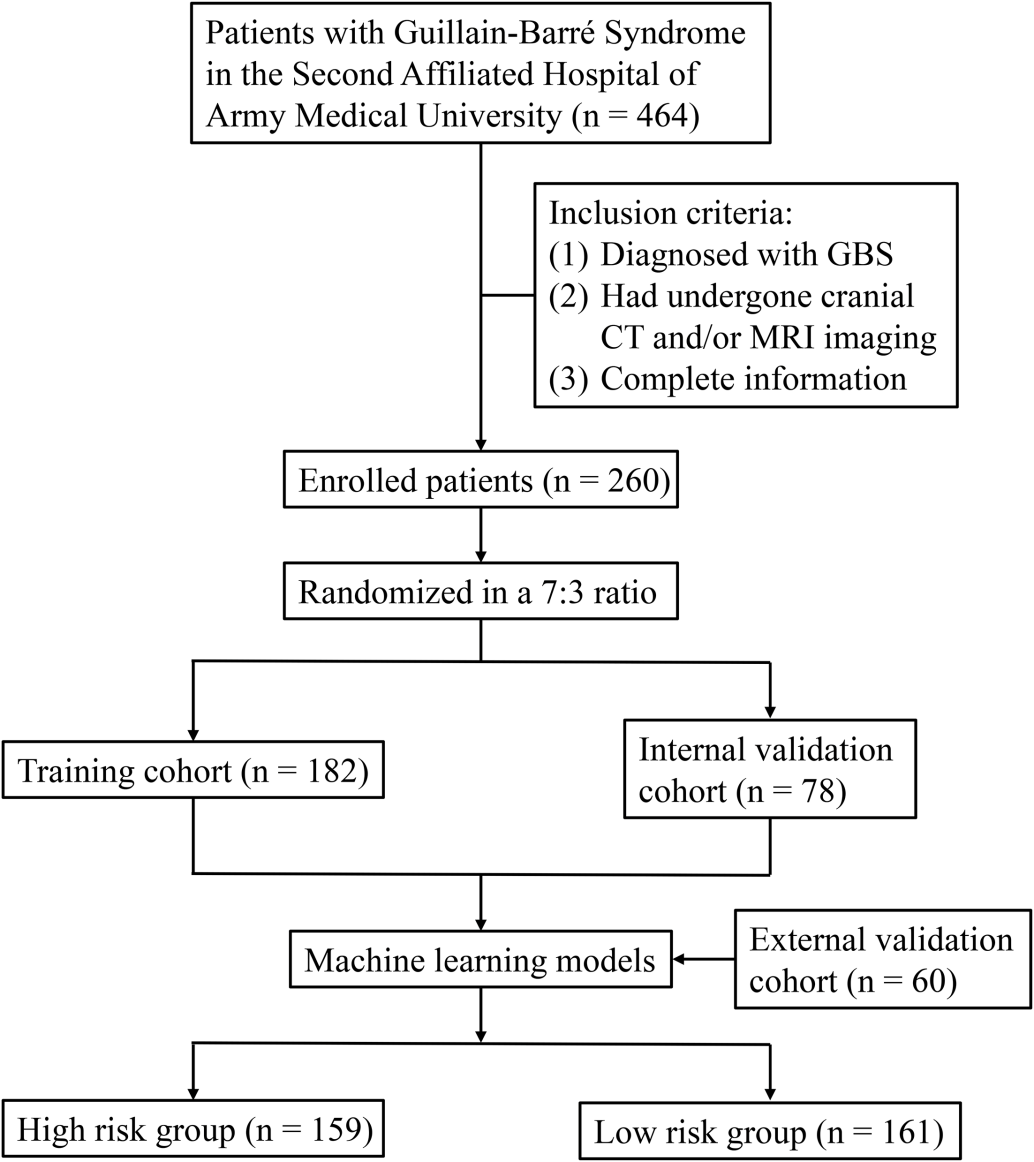
Flowchart outlining patient’s enrollment and study design.

This retrospective analysis utilized clinical data from patients diagnosed with GBS who received care at the Second Affiliated Hospital of Army Medical University (AMU) between January 1, 2015, and December 31, 2024. Eligibility criteria included: (1) confirmed GBS diagnosis; (2) availability of cranial CT and/or MRI imaging performed following symptom presentation; and (3) a threshold of missing data not exceeding 20% across collected parameters. For external validation purposes, a separate cohort of 60 GBS cases meeting identical inclusion criteria was selected from the First Affiliated Hospital of AMU during the same timeframe. **Figure 1** outlines the overall research design and methodological workflow. The investigation adhered to the ethical standards set forth in the Declaration of Helsinki and its subsequent revisions. Approval for the study protocol was granted by the local institutional review board, and the requirement for individual patient consent was waived due to the retrospective design. Reporting of methods and findings follows the STROBE guidelines.

### 2.2 Definition

According to guidelines from the World Health Organization (WHO), the American Heart Association/American Stroke Association (AHA/ASA), and the European Stroke Organization (ESO), stroke is defined as a clinical syndrome resulting from vascular causes leading to acute focal or global cerebral dysfunction, with neurological deficits persisting for ≥24 hours (or resulting in death within 24 hours) (15–18). Stroke is broadly classified into ischemic stroke and hemorrhagic stroke. Hemorrhagic stroke is further subdivided into intracerebral hemorrhage and subarachnoid hemorrhage. Ischemic stroke is categorized according to the TOAST classification into the following subtypes: large-artery atherosclerosis, cardioembolism, small-artery occlusion (lacunar infarction), stroke of other determined etiology, and stroke of undetermined etiology (19). In this study, the primary endpoint was defined as concomitant (coexisting) stroke identified during the index clinical episode in patients with GBS. Based on findings from head computed tomography (CT) and/or magnetic resonance imaging (MRI), patients were stratified into two groups: GBS with concomitant stroke and GBS without stroke. Because most patients were diagnosed with both conditions at admission and prior neuroimaging was unavailable, the temporal sequence between GBS and stroke could not be determined in the majority of cases. Therefore, stroke in the present study was interpreted as a coexisting condition.

### 2.3 Data collection

This study collected the following patient data: (1) Sociodemographic characteristics: sex, age, body mass index (BMI), smoking history, and alcohol consumption history; (2) Comorbidities: history of hypertension, diabetes mellitus, coronary heart disease, and dyslipidemia; (3) Disease-related history: type of antecedent infection, time of hospital admission, length of hospital stay, progression phase (defined as the interval from the onset of the first neurological symptoms to the plateau of clinical deficits) (20), stroke type, and stroke location; (4) Laboratory examinations: data collected at admission included cerebrospinal fluid white blood cell count (CSF-WBC, cells/μL), cerebrospinal fluid protein (CSF-Protein, g/L), white blood cell count (WBC, 10^9^/L), absolute neutrophil count (NEUT, 10^9^/L), absolute lymphocyte count (LYMPH, 10^9^/L), absolute monocyte count (MONO, 10^9^/L), neutrophil percentage (NEUT%, %), lymphocyte percentage (LYMPH%, %), monocyte percentage (MONO%, %), hemoglobin (Hb, g/L), red blood cell count (RBC, 10¹²/L), platelet count (PLT, 10^9^/L), sodium (Na, mmol/L), potassium (K, mmol/L), creatinine (Cr, μmol/L), estimated glomerular filtration rate (eGFR, mL/min/1.73 m²), uric acid (UA, μmol/L), alanine aminotransferase (ALT, U/L), aspartate aminotransferase (AST, U/L), high-density lipoprotein cholesterol (HDL-C, mmol/L), low-density lipoprotein cholesterol (LDL-C, mmol/L), creatine kinase (CK, U/L), creatine kinase-MB isoenzyme (CK-MB, U/L), prothrombin time (PT, s), international normalized ratio (INR), activated partial thromboplastin time (APTT, s), thrombin time (TT, s), fibrinogen (FIB, g/L), blood glucose (GLU, mmol/L), glycated hemoglobin (HbA1c, %), free triiodothyronine (FT3, pmol/L), free thyroxine (FT4, pmol/L), high-sensitivity thyroid-stimulating hormone (TSH, mIU/L), systemic inflammation composite index (AISI), lymphocyte to high-density lipoprotein cholesterol ratio (LHR), and monocyte to high-density lipoprotein cholesterol ratio (MHR); (5) Treatments during hospitalization: methylprednisolone, intravenous immunoglobulin (IVIg), plasma exchange (PE), and other therapies; (6) GBS-related scores: Guillain-Barré syndrome disability scale (GBS-DS) (21), inflammatory neuropathy cause and treatment disability scale (INCAT) (22), inflammatory Rasch-built overall disability scale (I-RODS) (23), and Medical Research Council muscle strength grading scale (MRC) (24) assessed at admission, discharge, and 3 months post-discharge. All laboratory variables used for model development, including APTT, RBC, GLU, and HbA1c, were obtained at hospital admission as part of the initial clinical evaluation. Given the retrospective design and the fact that most patients with cerebrovascular lesions were diagnosed with both GBS and stroke at admission, the temporal sequence between predictor measurement and stroke status could not be definitively established in the majority of cases. Therefore, these variables were interpreted as admission-time clinical markers associated with concomitant stroke rather than strictly pre-event predictors, and were used for admission-time individualized assessment rather than inference of a causal or strictly temporal relationship. All data were collected and archived by trained research staff. To ensure accuracy, an independent researcher performed logical checks and data re-evaluation. All examinations and tests were conducted at either the Second Affiliated Hospital or the First Affiliated Hospital of AMU.

### 2.4 Sample size

Sample size was determined by the events-per-variable (EPV) principle (25, 26). Because the prevalence of concomitant stroke in the GBS cohort reached roughly 50%, and nine predictors were pre-specified, an EPV of 10 yielded a minimum of 180 participants. Ultimately, 260 individuals were enrolled, exceeding the minimum sample size to enhance statistical power while improving the precision of analytical testing and the robustness of the predictive model.

### 2.5 Data preprocessing

To address potential bias from incomplete data, variables exhibiting missing values exceeding a 20% threshold were excluded during initial data processing. For remaining incomplete variables, multiple imputation was performed using R software (version 4.5.1) to generate plausible values for missing entries, enabling more robust subsequent analyses. All preprocessing procedures, including missing data imputation and feature selection, were conducted using the training set only, and the same transformations were subsequently applied to the internal and external validation cohorts to avoid information leakage. To enhance model generalizability and reduce overfitting risk, the study population was randomly partitioned into a training set (70%, n = 182) and an internal validation set (30%, n = 78) (27). Feature selection was conducted using least absolute shrinkage and selection operator (LASSO) regression, which identified nine predictor variables subsequently evaluated through ten-fold cross-validation. Seven machine learning algorithms were implemented: logistic regression (LR), decision tree, random forest (RF), extreme gradient boosting (XGBoost), light gradient boosting machine (LightGBM), support vector machine (SVM), and artificial neural network (ANN). Following grid search optimization of hyperparameters (28), models were trained using 5-fold cross-validation with resampling techniques. Detailed model specifications and hyperparameter settings are provided in **Supplementary Material 2**. Predictive performance was assessed on both internal and independent external validation cohorts. The external validation cohort was completely independent and was not involved in any stage of model development or parameter tuning. The area under the receiver operating characteristic curve (AUROC) served as the primary evaluation metric, supplemented by additional measures including accuracy, sensitivity, specificity, positive and negative predictive values (PPV, NPV), F1 score, and Cohen’s kappa. The optimal cutoff value was determined in the training set according to the maximum Youden index derived from the ROC curve and was subsequently applied unchanged to the validation cohorts. Decision curve analysis (DCA) quantified clinical utility across probability thresholds. All analyses were conducted in Python 3.10, with statistical significance defined as α = 0.05 (two-tailed).

### 2.6 SHAP interpretability analysis

Employing principles from cooperative game theory, the SHapley Additive exPlanations (SHAP) technique offers a model-agnostic methodology to elucidate prediction mechanisms across diverse machine learning architectures. This approach quantifies the marginal contribution of individual input variables to each prediction outcome, supporting concurrent interpretation at both instance-specific and population-wide levels. By deconstructing the decision logic embedded within black-box models, the framework significantly improves operational transparency and model interpretability (29). In the present study, SHAP analysis was used to interpret the contribution of variables to the identification of concomitant stroke in GBS patients.

### 2.7 Statistical analysis

Continuous measures exhibiting normal distributions are reported as mean ± standard deviation; non-normally distributed variables are expressed as median with interquartile range. Categorical data are presented as frequency counts and percentages. Group comparisons were performed using statistical methods appropriate to data distribution patterns: Student’s t-test for normally distributed continuous variables, Wilcoxon rank-sum test for non-parametric data, and chi-square or Fisher’s exact test for categorical variables. A two-sided significance level of p < 0.05 was applied for all analyses.

Based on the model-derived cutoff determined in the training set, subjects were classified into high-risk and low-risk subgroups. Short-term prognostic differences between these strata were subsequently evaluated. Longitudinal changes in GBS disability scores were analyzed using repeated-measures ANOVA (rmANOVA), examining the effects of risk category (high vs. low), concomitant stroke status (present vs. absent), and treatment type (methylprednisolone, IVIg, plasma exchange, or other therapies). All analytical procedures and visualization tasks were implemented using SPSS (v29.0), R (v4.5.1), Python (v3.10), and GraphPad Prism (v10.4.1).

## 3 Results

### 3.1 Patient characteristics

The final analytical dataset incorporated 48 clinically relevant variables selected for modeling. Detailed characterization of missing data distributions and patterns across these parameters is documented in **Supplementary Material 1**.

This investigation initially identified 464 individuals diagnosed with GBS who received treatment at the Second Affiliated Hospital of AMU from January 1, 2015, to December 31, 2024. After implementing the predefined inclusion criteria, a total of 260 subjects were included in the final analytical cohort. Within this cohort, 130 patients (50.0%) had concomitant stroke. Among these 130 patients, 121 (93.08%) were concurrently diagnosed with GBS and stroke at admission, and no prior neuroimaging was available to determine the temporal sequence between the two conditions; therefore, these cases were classified as concomitant stroke within the same clinical episode, whereas 9 (6.92%) had documented stroke before the onset of GBS. Demographic data indicated a male predominance (60.38%), and the median patient age was 52.00 years (IQR: 39.00–64.25). Regarding behavioral factors, 70.38% of participants reported a history of tobacco use, and 83.08% had a record of alcohol consumption. The median body mass index (BMI) was 22.96 kg/m² (IQR: 21.03–24.95). Common comorbidities included hypertension (25.00%), diabetes mellitus (15.00%), hyperlipidemia (23.46%), and coronary heart disease (3.85%). Prior to onset, upper respiratory tract infection (URTI) was documented in 36.92% of cases, whereas no clear precipitating factors were identified in 48.46% of patients. The median disease progression phase for GBS was 16.00 days (IQR: 10.00–25.00). Comparative analyses of baseline characteristics between the concomitant-stroke and non-concomitant-stroke subgroups, as well as between the training and internal validation cohorts, are summarized in **Table 1**.

**Table 1:** Patient’s demographics, comorbidities, medical history and tests.

Characteristics	Whole	Concomitant stroke		P	Cohort		P
Groups	Patients	No	Yes		Training	Internal validation	
n	260	130	130		182	78	
Demographics							
Gender (male/female, %)	157/103 (60.38/39.62)	75/55 (57.69/42.31)	82/48 (63.08/36.92)	0.447	113/69 (62.09/37.91)	44/34 (56.41/43.59)	0.472
Age (median [IQR], years)	52.00 [39.00, 64.25]	41.50 [32.00, 52.00]	60.50 [51.25, 69.00]	<0.001	53.00 [40.00, 66.00]	51.00 [36.25, 62.50]	0.146
Smoking history (no/yes, %)	183/77 (70.38/29.62)	97/33 (74.62/25.38)	86/44 (66.15/33.85)	0.174	128/54 (70.33/29.67)	55/23 (70.51/29.49)	1.000
Alcohol intake history (no/yes, %)	216/44 (83.08/16.92)	117/13 (90.00/10.00)	99/31 (76.15/23.85)	0.005	153/29 (84.07/15.93)	63/15 (80.77/19.23)	0.639
BMI (median [IQR], kg/m^2^)	22.96 [21.03, 24.95]	22.49 [20.83, 24.56]	23.35 [21.23, 25.18]	0.154	23.13 [21.09, 25.04]	22.88 [20.91, 24.60]	0.859
Comorbidities							
Hypertension (no/yes, %)	195/65 (75.00/25.00)	119/11 (91.54/8.46)	76/54 (58.46/41.54)	<0.001	139/43 (76.37/23.63)	56/22 (71.79/28.21)	0.532
Diabetes (no/yes, %)	221/39 (85.00/15.00)	119/11 (91.54/8.46)	102/28 (78.46/21.54)	0.005	152/30 (83.52/16.48)	69/9 (88.46/11.54)	0.404
Coronary heart disease (no/yes, %)	250/10 (96.15/3.85)	128/2 (98.46/1.54)	122/8 (93.85/6.15)	0.107	174/8 (95.60/4.40)	76/2 (97.44/2.56)	0.725
Hyperlipidemia (no/yes, %)	199/61 (76.54/23.46)	105/25 (80.77/19.23)	94/36 (72.31/27.69)	0.143	139/43 (76.37/23.63)	60/18 (76.92/23.08)	1.000
Medical history							
Preceding infection (URTI/diarrhea/Other/ No apparent precipitating factor, %)	96/18/20/126 (36.92/6.92/7.69/48.46)	56/10/8/56 (43.08/7.69/6.15/43.08)	40/8/12/70 (30.77/6.15/9.23/53.85)	0.155	68/13/11/90 (37.36/7.14/6.04/49.45)	28/5/9/36 (35.90/6.41/11.54/46.15)	0.505
Progressive stage (median [IQR], days)	16.00 [10.00, 25.00]	16.00 [10.25, 26.00]	15.50 [10.00, 21.00]	0.556	16.00 [10.00, 25.75]	14.00 [10.25, 22.75]	0.454
Tests							
CSF-WBC (median [IQR], cells/μL)	2.50 [1.00, 5.00]	2.00 [1.00, 5.00]	3.00 [1.00, 4.00]	0.720	2.00 [1.00, 4.00]	3.00 [1.00, 5.00]	0.250
CSF-Protein (median [IQR], CSF-Protein, g/L)	0.67 [0.36, 1.13]	0.70 [0.36, 1.25]	0.63 [0.36, 1.07]	0.445	0.65 [0.36, 1.11]	0.72 [0.36, 1.29]	0.538
WBC (median [IQR], 10^9^/L)	7.44 [6.05, 9.80]	7.58 [6.00, 9.76]	7.14 [6.06, 9.81]	0.425	7.44 [6.18, 9.81]	7.47 [5.88, 9.59]	0.769
NEUT (median [IQR], 10^9^/L)	5.08 [3.84, 7.41]	5.26 [3.85, 7.22]	4.97 [3.81, 7.71]	0.825	5.14 [3.88, 7.50]	5.02 [3.81, 7.13]	0.819
LYMPH (median [IQR], 10^9^/L)	1.54 [1.13, 2.08]	1.63 [1.18, 2.17]	1.40 [1.05, 1.94]	0.048	1.54 [1.12, 2.00]	1.53 [1.14, 2.12]	0.736
MONO (median [IQR], 10^9^/L)	0.50 [0.39, 0.66]	0.49 [0.39, 0.64]	0.52 [0.39, 0.70]	0.328	0.52 [0.40, 0.67]	0.48 [0.36, 0.66]	0.362
NEUT% (median [IQR], %)	69.40 [60.90, 78.82]	68.70 [60.75, 78.25]	70.45 [62.80, 79.27]	0.552	69.65 [59.30, 79.12]	69.10 [62.80, 77.28]	0.796
LYMPH% (median [IQR], %)	21.45 [13.40, 28.80]	22.35 [15.03, 30.05]	19.60 [13.12, 27.80]	0.133	21.15 [13.40, 29.73]	21.80 [14.43, 27.75]	0.773
MONO% (median [IQR], %)	6.70 [5.10, 8.60]	6.25 [4.80, 8.15]	7.00 [5.60, 8.80]	0.061	6.60 [5.10, 8.70]	6.90 [5.10, 8.00]	0.732
Hb (median [IQR], g/L)	134.00 [124.00, 147.00]	138.00 [126.25, 148.00]	133.00 [122.25, 144.50]	0.046	135.00 [124.25, 149.75]	133.00 [121.25, 141.75]	0.097
RBC (median [IQR], 10¹²/L)	4.52 [4.11, 4.89]	4.60 [4.14, 4.94]	4.42 [4.02, 4.85]	0.071	4.54 [4.14, 4.90]	4.46 [4.06, 4.85]	0.278
PLT (median [IQR], 10^9^/L)	230.00 [184.00, 292.00]	242.50 [189.00, 291.00]	223.00 [182.25, 291.50]	0.249	226.00 [180.50, 287.75]	243.50 [192.25, 309.00]	0.091
Na (median [IQR], mmol/L)	138.35 [135.98, 140.00]	138.35 [136.12, 140.00]	138.30 [135.83, 140.20]	0.940	138.50 [136.20, 140.20]	137.85 [135.35, 139.52]	0.097
K (median [IQR], mmol/L)	3.92 [3.70, 4.17]	3.96 [3.73, 4.22]	3.88 [3.64, 4.14]	0.115	3.92 [3.70, 4.16]	3.94 [3.70, 4.25]	0.721
Cr (median [IQR], μmol/L)	61.25 [53.00, 74.00]	59.40 [51.02, 72.10]	63.25 [54.18, 75.80]	0.026	61.35 [53.90, 72.97]	61.25 [50.95, 78.50]	0.943
eGFR (median [IQR], mL/min/1.73 m²)	104.00 [93.00, 117.46]	112.72 [101.00, 124.28]	98.56 [88.00, 106.00]	<0.001	102.20 [93.00, 117.82]	106.00 [95.15, 116.97]	0.383
UA (median [IQR], μmol/L)	296.50 [232.75, 362.95]	287.35 [238.48, 372.92]	301.20 [226.25, 343.25]	0.849	298.50 [237.27, 355.83]	287.85 [220.38, 366.38]	0.975
ALT (median [IQR], U/L)	25.00 [16.50, 42.52]	27.00 [17.33, 44.22]	22.90 [15.85, 37.80]	0.166	25.15 [16.52, 42.48]	23.65 [15.70, 43.38]	0.864
AST (median [IQR], U/L)	22.70 [17.67, 32.45]	23.20 [18.75, 33.00]	22.00 [16.52, 31.75]	0.212	22.85 [17.47, 31.98]	22.55 [18.05, 33.68]	0.651
HDL-C (median [IQR], mmol/L)	1.12 [0.92, 1.32]	1.15 [0.93, 1.38]	1.06 [0.90, 1.28]	0.076	1.11 [0.92, 1.30]	1.13 [0.92, 1.34]	0.506
LDL-C (mean (SD), mmol/L)	2.67 (0.80)	2.77 (0.77)	2.58 (0.83)	0.063	2.67 (0.80)	2.69 (0.82)	0.826
CK (median [IQR], U/L)	73.00 [47.90, 134.02]	73.00 [45.73, 133.12]	74.70 [49.27, 133.25]	0.900	69.75 [44.47, 117.07]	81.85 [52.17, 157.23]	0.158
CK-MB (median [IQR], U/L)	13.00 [9.00, 17.20]	12.35 [9.00, 16.45]	13.00 [9.00, 18.00]	0.479	13.00 [9.00, 18.00]	13.00 [9.12, 16.00]	0.885
PT (median [IQR], s)	10.80 [10.20, 11.53]	10.80 [10.20, 11.50]	10.70 [10.22, 11.60]	0.878	10.80 [10.30, 11.50]	10.70 [10.12, 11.57]	0.554
INR (median [IQR])	0.96 [0.90, 1.04]	0.96 [0.90, 1.04]	0.95 [0.90, 1.05]	0.956	0.96 [0.91, 1.04]	0.94 [0.89, 1.04]	0.237
APTT (median [IQR], s)	28.75 [25.80, 31.42]	28.05 [25.55, 30.30]	29.70 [26.30, 32.62]	0.011	28.75 [25.72, 31.40]	28.80 [26.18, 31.80]	0.738
TT (median [IQR], s)	15.70 [13.50, 18.20]	15.65 [13.53, 18.35]	15.80 [13.50, 17.78]	0.800	15.55 [13.33, 18.05]	16.30 [13.65, 18.62]	0.221
FIB (median [IQR], g/L)	3.06 [2.58, 3.71]	2.93 [2.45, 3.50]	3.21 [2.73, 3.84]	0.022	3.11 [2.64, 3.74]	2.99 [2.42, 3.65]	0.208
GLU (median [IQR], mmol/L)	5.47 [4.81, 6.63]	5.24 [4.56, 6.17]	5.76 [5.10, 7.19]	<0.001	5.46 [4.86, 6.62]	5.47 [4.78, 6.67]	0.888
HbA1c (median [IQR], %)	5.80 [5.40, 6.20]	5.60 [5.20, 5.90]	5.90 [5.60, 6.38]	<0.001	5.80 [5.40, 6.20]	5.75 [5.40, 6.00]	0.581
FT3 (median [IQR], pmol/L)	4.30 [3.59, 4.90]	4.42 [3.70, 4.98]	4.12 [3.46, 4.72]	0.055	4.31 [3.45, 4.90]	4.28 [3.71, 4.84]	0.542
FT4 (median [IQR], pmol/L)	16.17 [13.76, 18.52]	16.67 [13.92, 18.81]	15.52 [13.56, 18.14]	0.183	16.29 [13.78, 18.61]	15.83 [13.66, 18.23]	0.458
TSH (median [IQR], mIU/L)	1.77 [1.00, 2.75]	1.87 [1.14, 2.58]	1.72 [0.96, 2.89]	0.669	1.80 [1.02, 2.63]	1.64 [1.00, 2.98]	0.919
AISI (median [IQR])	549.13 [326.63, 963.66]	547.95 [320.77, 976.87]	553.50 [337.01, 908.93]	0.922	564.46 [335.46, 943.57]	526.36 [264.72, 1019.97]	0.893
LHR (median [IQR])	1.41 [0.98, 1.98]	1.45 [1.00, 2.13]	1.39 [0.95, 1.95]	0.371	1.42 [0.99, 1.99]	1.37 [0.93, 1.97]	0.769
MHR (median [IQR])	0.49 [0.32, 0.69]	0.45 [0.28, 0.68]	0.52 [0.34, 0.72]	0.145	0.49 [0.34, 0.68]	0.44 [0.28, 0.72]	0.564

IQR, interquartile range; BMI, body mass index; URTI, upper respiratory tract infection; CSF-WBC, cerebrospinal fluid white blood cell count; CSF-Protein, cerebrospinal fluid total protein; WBC, white blood cell count; NEUT, absolute neutrophil count; LYMPH, absolute lymphocyte count; MONO, absolute monocyte count; NEUT%, neutrophil percentage; LYMPH%, lymphocyte percentage; MONO%, monocyte percentage; Hb, hemoglobin; RBC, red blood cell count; PLT, platelet count; Na, serum sodium; K, serum potassium; Cr, serum creatinine; eGFR, estimated glomerular filtration rate; UA, uric acid; ALT, alanine aminotransferase; AST, aspartate aminotransferase; HDL-C, high-density lipoprotein cholesterol; LDL-C, low-density lipoprotein cholesterol; SD, standard deviation; CK, creatine kinase; CK-MB, creatine kinase-mb isoenzyme; PT, prothrombin time; INR, international normalized ratio; APTT, activated partial thromboplastin time; TT, thrombin time; FIB, fibrinogen; GLU, plasma glucose; HbA1c, glycated hemoglobin A1c; FT3, free triiodothyronine; FT4, free thyroxine; TSH, thyroid-stimulating hormone; AISI, aggregate index of systemic inflammation; LHR, lymphocyte-to-HDL-C ratio; MHR, monocyte-to-HDL-C ratio.

**Figure 2:**
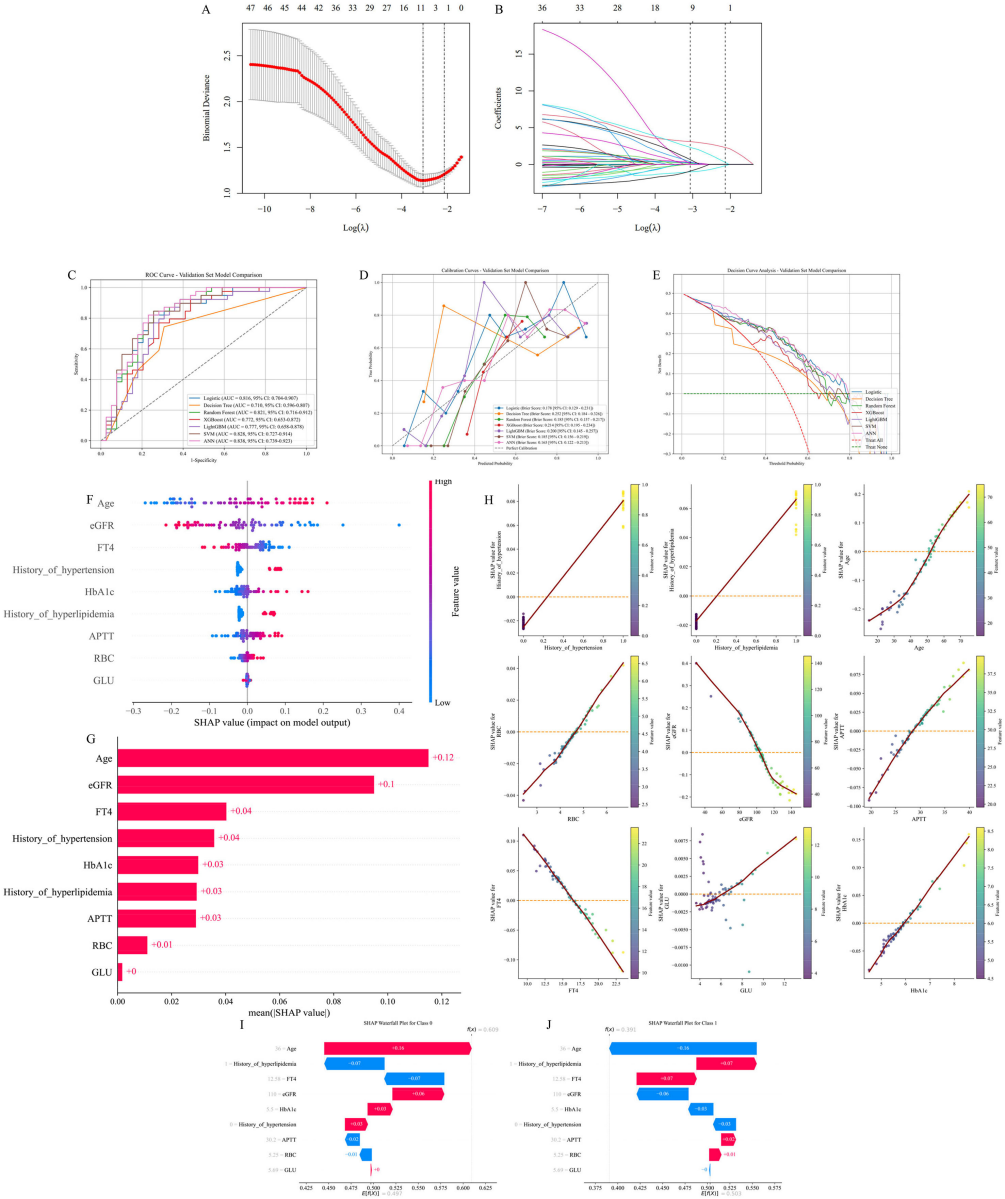
Machine learning model. **(A)** LASSO regression model factor selection; **(B)** LASSO regression model screening variable trajectories; **(C)** ROC Curves for the internal validation cohort; **(D)** Calibration Curve for the internal validation cohort; **(E)** Decision Curve Analysis for the internal validation cohort; **(F)** Characteristic attributes in SHAP; **(G)** Importance ranking plot of features. **(H)** Scatter plots of features; **(I, J)**. Interpretability analysis of 2 independent samples.

Variable selection was performed using LASSO regression. Following 10-fold cross-validation, 13 potential predictors were initially identified. Subsequently, four variables were excluded based on clinical relevance and expert judgment, resulting in a final set of nine predictors for model construction: history of hypertension, history of hyperlipidemia, age, RBC, eGFR, APTT, FT4, GLU, and HbA1c. The variable selection process and coefficient shrinkage paths are illustrated in **Figures 2A, B**.

### 3.2 Multimodel integrated analysis for classification

Seven machine learning models were developed and applied to identify concomitant stroke in the study cohort comprising individuals diagnosed with GBS. Following the training phase and optimization of hyperparameters, the ideal configuration for each model was identified, with full specifications provided in **Supplementary Material 2**. Comprehensive evaluation results for all algorithms, incorporating essential performance indicators, are presented in **Figures 2C-E** and further elaborated in **Supplementary Materials 3**–**7**.

After consolidating predictive features from the integrated training, internal validation, and external validation datasets, the ANN demonstrated the highest level of predictive accuracy and robustness for identifying concomitant stroke in GBS patients. The ANN consistently exhibited strong discriminatory power across all evaluated cohorts. In the internal validation set, it achieved an AUROC of 0.838 (95% CI: 0.739–0.923), accompanied by the following performance metrics: accuracy 0.782, precision 0.789, sensitivity 0.769, specificity 0.795, F1 score 0.779, Cohen’s kappa 0.564, PPV 0.789, and NPV 0.775. Within the training cohort, the model obtained an AUROC of 0.849 (95% CI: 0.791–0.898), with corresponding values for accuracy 0.764, precision 0.750, sensitivity 0.791, specificity 0.736, F1 score 0.770, Cohen’s kappa 0.527, PPV 0.750, and NPV 0.779. Similarly, in the external test cohort, the ANN maintained commendable performance, producing an AUROC of 0.782 (95% CI: 0.652–0.892) along with accuracy 0.733, precision 0.760, sensitivity 0.655, specificity 0.806, F1 0.703, Cohen’s kappa 0.464, PPV 0.760, and NPV 0.714. While other machine learning algorithms also showed substantial predictive performance, the ANN was selected as the optimal model owing to its superior stability and consistent predictive outcomes across heterogeneous datasets. These results indicate consistent model performance across the training, internal validation, and independent external validation datasets, supporting its generalizability.

### 3.3 Interpretability of the model

**Figures 2F–H** displays SHAP (SHapley Additive exPlanations) summary plots, providing interpretable insights into the ANN model’s predictive logic for concomitant stroke in GBS patients. Among the evaluated predictors, age, eGFR, FT4, and history of hypertension were determined to have the strongest influence on the identification of concomitant stroke. Increased values of age, HbA1c, APTT, RBC, GLU, as well as history of hypertension or history of hyperlipidemia, showed positive associations with concomitant stroke. In contrast, elevated eGFR and FT4 levels exhibited protective effects, corresponding to decreased likelihood of concomitant stroke. Additionally, **Figures 2I, J** presents two representative clinical cases to demonstrate the model’s local interpretability at the individual prediction level, complementing the global interpretation provided by the SHAP summary plots. To facilitate practical application of the final ANN model, we implemented a deployable web-based platform using Streamlit (https://gbs-concomitant-stroke-assessment-7uybrpemwruzc6xlzn4vsg.streamlit.app/) and made the source code publicly available on GitHub. The web application provides an online tool for individualized assessment of concomitant stroke in patients with GBS. By entering admission-time clinical variables into the designated input fields, users can conveniently obtain the corresponding model-estimated probability and assessment result.

### 3.4 Sensitivity analysis

Sensitivity analysis excluding the 9 patients with pre-existing stroke yielded improved performance for the ANN model. After retraining the model using only the 121 patients in whom the temporal sequence between GBS and stroke could not be determined, the internal validation AUROC was 0.877 (95% CI: 0.781–0.953), which was comparable to the original result of 0.838. In the external validation cohort, where no patients had documented pre-existing stroke, the model also remained stable, with an AUROC of 0.843. These findings suggest that the model performance was not driven by patients with known prior stroke and supports the robustness of the model in identifying concomitant stroke.

### 3.5 Prognostic comparison

**Figure 3:**
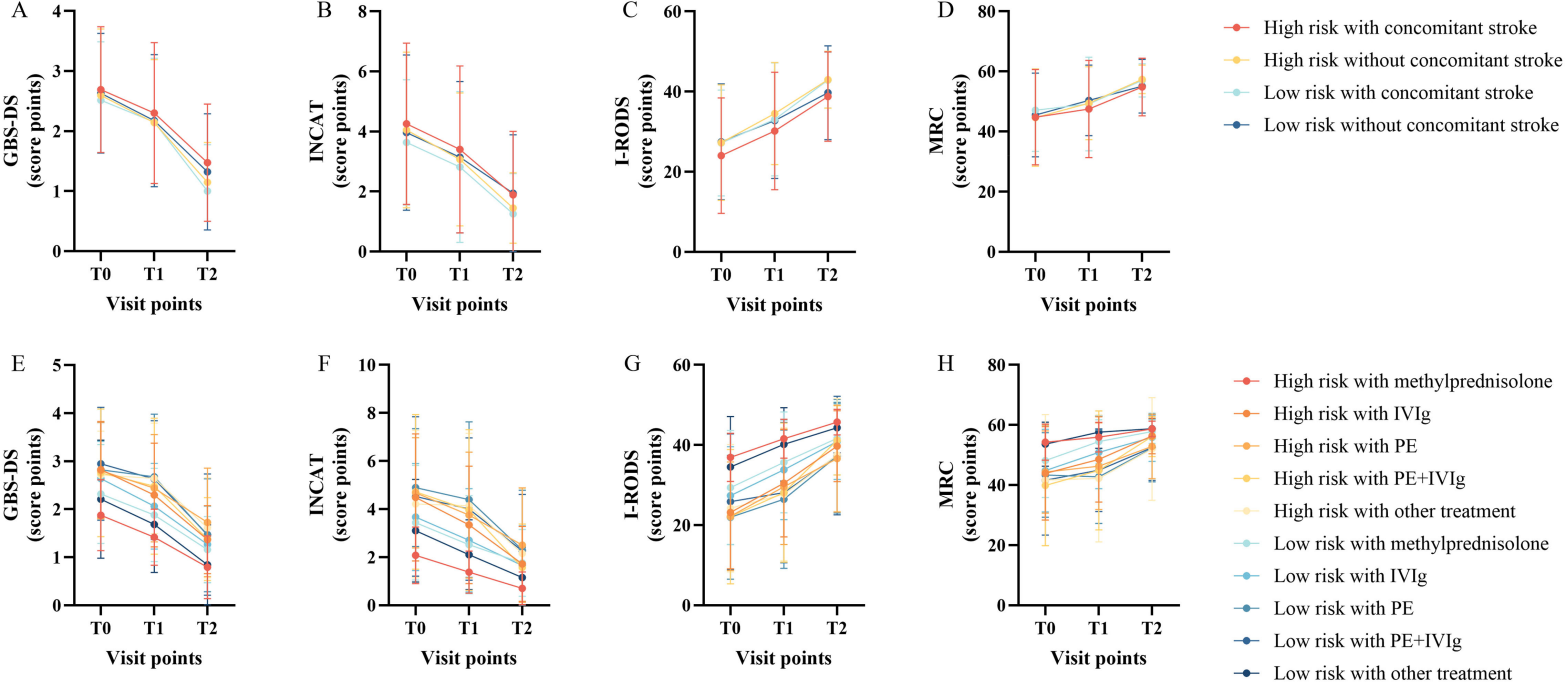
Line graph of GBS scores. T0, at admission; T1, at discharge; T2, 3 months post-discharge. **(A)** Changes in GBS-DS scores in patients with and without concomitant stroke stratified by risk level; **(B)** Changes in INCAT scores in patients with and without concomitant stroke stratified by risk level; **(C)** Changes in I-RODS scores in patients with and without concomitant stroke stratified by risk level; **(D)** Changes in MRC scores in patients with and without concomitant stroke stratified by risk level; **(E)** Changes in GBS-DS scores in patients receiving different treatments, stratified by risk level; **(F)** Changes in INCAT scores in patients receiving different treatments, stratified by risk level; **(G)** Changes in I-RODS scores in patients receiving different treatments, stratified by risk level; **(H)** Changes in MRC scores in patients receiving different treatments, stratified by risk level.

**Figure 4:**
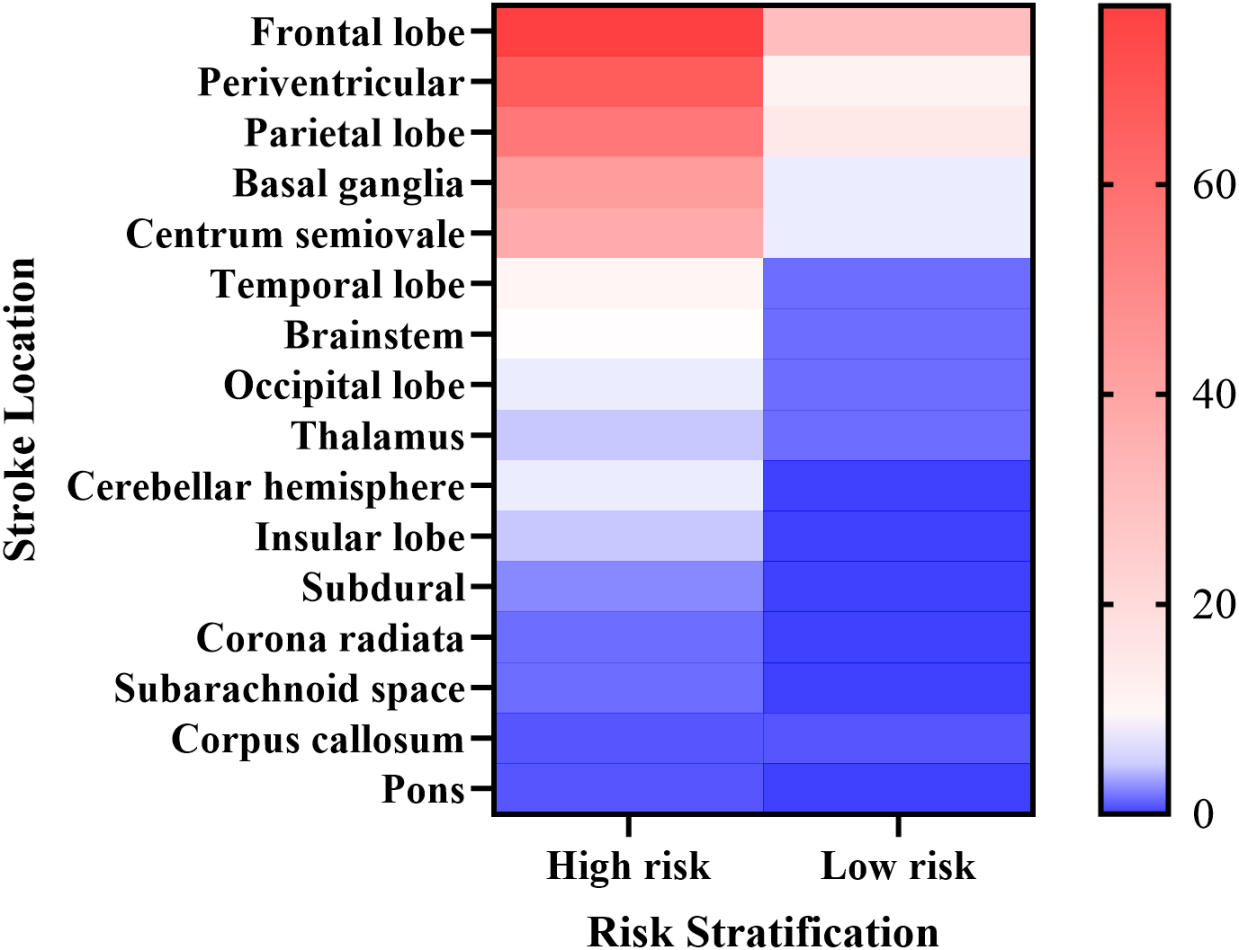
Stroke location based on risk stratification.

Based on the optimal cutoff probability (0.450) determined by the ANN model, patients from the training, internal validation, and external validation cohorts were reclassified. Subjects with a predicted probability score below 0.450 were assigned to the low-risk category (n=152, 47.5%), while those scoring 0.450 or above were placed in the high-risk category (n=168, 52.5%). The progression of functional outcomes across these risk strata is visualized in **Figure 3**. A rmANOVA incorporating Greenhouse–Geisser correction was conducted to assess the longitudinal impacts of risk category, concomitant stroke status, and treatment type on functional recovery; detailed results are presented in **Table 2**. All functional assessment instruments showed a statistically significant main effect for time (p<0.001), indicating considerable overall functional improvement between hospital admission and the 3-month follow-up evaluation. No statistically significant interaction effects were observed, however, for any two-way interactions (Time×Risk, Time×Stroke, Time×Treatment) or three-way interactions across the scales (p>0.05). Additionally, anatomical localization of stroke lesions was examined among patients with concomitant stroke, stratified by risk category (**Figure 4**). The most commonly affected neuroanatomical regions included the frontal lobe, periventricular white matter, parietal lobe, basal ganglia, and centrum semiovale.

**Table 2:** Results of repeated-measures ANOVA (Greenhouse–Geisser correction).

Source	F (df, df_error)	P	Partial η²
GBS-DS			
Time	270.75(1.86, 563.37)	<0.001	0.472
Time×RiskGroup	1.08(1.86, 563.37)	0.337	0.004
Time×Stroke	0.11(1.86, 563.37)	0.882	<0.001
Time×RiskGroup×Stroke	1.62(1.86, 563.37)	0.201	0.005
INCAT			
Time	180.7(1.75, 528.65)	<0.001	0.374
Time×RiskGroup	0.18(1.75, 528.65)	0.803	0.001
Time×Stroke	0.12(1.75, 528.65)	0.861	<0.001
Time×RiskGroup×Stroke	0.43(1.75, 528.65)	0.621	0.001
I-RODS			
Time	217.8(1.74, 526.99)	<0.001	0.418
Time×RiskGroup	0.55(1.74, 526.99)	0.555	0.002
Time×Stroke	0.10(1.74, 526.99)	0.876	<0.001
Time×RiskGroup×Stroke	0.67(1.74, 526.99)	0.494	0.002
MRC			
Time	133.4(1.60, 483.28)	<0.001	0.306
Time×RiskGroup	0.17(1.60, 483.28)	0.798	0.001
Time×Stroke	1.19(1.60, 483.28)	0.298	0.004
Time×RiskGroup×Stroke	0.43(1.60, 483.28)	0.607	0.001
GBS-DS			
Time	275.1(1.85, 548.82)	<0.001	0.481
Time×RiskGroup	2.21(1.85, 548.82)	0.115	0.007
Time×Treatment	0.93(7.39, 548.82)	0.488	0.012
Time×RiskGroup×Treatment	0.93(7.39, 548.82)	0.49	0.012
INCAT			
Time	187.2(1.74, 515.58)	<0.001	0.387
Time×RiskGroup	0.05(1.74, 515.58)	0.933	<0.001
Time×Treatment	1.65(6.94, 515.58)	0.119	0.022
Time×RiskGroup×Treatment	1.13(6.94, 515.58)	0.343	0.015
I-RODS			
Time	215.8(1.74, 516.06)	<0.001	0.421
Time×RiskGroup	0.51(1.74, 516.06)	0.576	0.002
Time×Treatment	1.23(6.95, 516.06)	0.284	0.016
Time×RiskGroup×Treatment	0.93(6.95, 516.06)	0.485	0.012
MRC			
Time	132.5(1.58, 468.86)	<0.001	0.308
Time×RiskGroup	0.38(1.58, 468.86)	0.634	0.001
Time×Treatment	2.38(6.32, 468.86)	0.026	0.031
Time×RiskGroup×Treatment	1.52(6.32, 468.86)	0.167	0.02

## 4 Discussion

This investigation leveraged clinical records from two tertiary medical centers to determine pivotal predictors linked to concomitant stroke in individuals diagnosed with GBS. Seven machine learning approaches were applied to construct predictive frameworks. Comparative evaluation indicated that the ANN achieved the best performance for identifying concomitant stroke, with an AUC value of 0.838. Dimensionality reduction was performed using LASSO regression combined with tenfold cross-validation, yielding nine clinically relevant predictors. Within this set, increased age, history of hypertension, HbA1c, history of hyperlipidemia, APTT, RBC, and GLU were associated with a higher likelihood of concomitant stroke. Conversely, eGFR and FT4 concentrations showed protective associations. SHAP methodology further highlighted age, eGFR, FT4, and history of hypertension as the four foremost factors influencing risk stratification. All laboratory variables included in the model, such as APTT, RBC, GLU, and HbA1c, were obtained at hospital admission and should therefore be interpreted as admission-time clinical markers associated with concomitant stroke.

Previous investigations provide a robust foundation for the current study. Age, as a primary non-modifiable predictor, often exerts greater influence than other static risk factors (30). Advanced age is directly associated with increased atherosclerotic burden, impaired cerebrovascular autoregulation, and endothelial dysfunction (31–33). In patients with GBS, advanced age may indicate diminished physiological reserve and a more vulnerable blood-brain barrier, thereby increasing susceptibility to cerebrovascular events during acute immune-inflammatory challenges. Reduced eGFR serves as a sensitive marker for systemic vasculopathy (including cerebral small vessel disease) and endothelial dysfunction (34, 35). During the acute phase of GBS, impaired eGFR may exacerbate systemic inflammation and electrolyte disturbances, collectively promoting a hypercoagulable state and an increased likelihood of concomitant cerebrovascular lesions. Thyroid dysfunction is linked to dyslipidemia, endothelial dysfunction, and low-grade inflammatory states (36). GBS, as a significant physiological stressor, may disrupt thyroid hormone levels via the hypothalamic-pituitary-thyroid axis (37, 38); this metabolic-immune dysregulation may compromise cerebrovascular stability. Hypertension represents the most well-characterized risk factor for stroke, particularly lacunar infarction (39, 40). It directly damages vascular endothelium through mechanical stress and accelerates atherosclerosis (41). In GBS patients, autonomic dysfunction during the acute phase may cause dramatic blood pressure fluctuations (42), substantially increasing stroke susceptibility. Elevated HbA1c levels reflect suboptimal long-term glycemic control, indicating chronic inflammation, oxidative stress, and accumulation of advanced glycation end products, all of which contribute to vascular endothelial injury and atherosclerosis (43, 44). Dyslipidemia, particularly elevated low-density lipoprotein cholesterol, plays a central role in the development and progression of atherosclerotic plaques (45). It acts synergistically with hypertension and hyperglycemia, significantly increasing the risk of large-artery and cardioembolic infarction (46). While prolonged APTT is usually associated with bleeding tendency, it may also reflect the presence of lupus anticoagulant or other antiphospholipid antibodies in a prothrombotic setting (47, 48). GBS, being an autoimmune disorder, may trigger transient autoantibody production (49), potentially affecting coagulation and increasing thrombosis risk. Abnormalities in RBC count, morphology, or function can influence blood viscosity and oxygen-carrying capacity; elevated RBC levels may increase blood hyperviscosity, promoting thrombus formation (50). Furthermore, severe stress responses during the acute phase of GBS (e.g., catecholamine and cortisol release) may induce stress hyperglycemia (51). Acute hyperglycemia exhibits direct neurotoxicity, exacerbating post-ischemic brain injury and amplifying systemic inflammatory responses (52). However, because most patients in this retrospective cohort were diagnosed with GBS and stroke during the same clinical episode, these variables cannot be regarded as definitively pre-stroke predictors in a strict temporal sense. These findings may help explain why the selected variables were associated with concomitant stroke in our cohort. In addition, the SHAP analysis provided interpretable evidence at both the global and individual levels, thereby improving the transparency of the model output.

Using the ANN model, patients diagnosed with GBS were effectively categorized into two prognostic groups: a high-risk cohort (52.5%) and a low-risk cohort (47.5%) for concomitant stroke. For those classified as high-risk, clinicians might consider intensified immunomodulatory treatments—such as IVIg or PE—as well as closer monitoring of respiratory, swallowing, and motor functions. Longitudinal evaluation of functional outcomes revealed marked neurological improvement from admission to the three-month follow-up across all GBS patients, consistent with the typical recovery course of GBS and supporting the overall effectiveness of conventional interventions. Although the rate of functional recovery did not significantly differ between risk groups, individuals in the high-risk category presented with more severe neurological impairments at baseline. Importantly, despite matching the recovery trajectory of the low-risk group, high-risk patients showed consistently lower absolute function scores at each evaluation interval (admission, discharge, and three months post-discharge), which can be explained by their poorer initial clinical status. These findings suggest that risk stratification captures cross-sectional functional severity rather than differential recovery speed. Further exploration of interaction effects will necessitate larger sample sizes, highlighting an important avenue for subsequent research. Among GBS patients with concomitant stroke, frequent frontoparietal lesions may disrupt motor planning and fine motor control, while ischemic involvement of the basal ganglia and centrum semiovale may compound peripheral nerve damage, potentially worsening motor impairment and partly accounting for the reduced functional scores seen in this patient subgroup.

This study bridges an important knowledge gap through the application of machine learning techniques to elucidate the association between GBS and concomitant stroke. However, several methodological limitations should be acknowledged. First, because this was a retrospective study and most patients were diagnosed with GBS and stroke during the same clinical episode, the temporal sequence between the two conditions could not be definitively established in the majority of cases. Accordingly, all laboratory predictors identified in the present study should be interpreted as admission-time variables associated with concomitant stroke, rather than as strictly pre-event predictors collected before stroke occurrence. The model should therefore be viewed as a tool for admission-time identification and individualized assessment of concomitant stroke, rather than for prediction of future stroke occurrence after GBS onset. Second, the restricted sample size may have reduced the ability to identify complex effect modifications, a challenge frequently encountered in research on uncommon neurological conditions like GBS. Therefore, subsequent studies would benefit from the inclusion of larger participant groups to strengthen the reliability and external validity of the results. Third, owing to the retrospective nature of the investigation, prognostic evaluation was necessarily limited to GBS disability scores documented at the 3-month post-discharge interval—the most systematically recorded follow-up time point available. Although this affords valuable insight into short-term functional outcomes, it precludes assessment of potential disparities in longer-term recovery trajectories (for instance, at 6 or 12 months) across different patient subgroups. Finally, while the developed model demonstrated acceptable discriminative performance in an external validation sample, its broader clinical applicability and translational utility still require confirmation in larger prospective multicenter studies, particularly with respect to risk of bias, applicability, and overall study quality (53).

## 5 Conclusion

In summary, this investigation developed and assessed seven machine learning approaches to estimate the likelihood of concomitant stroke in individuals diagnosed with GBS. The ANN outperformed other models, demonstrating higher predictive accuracy and greater robustness, thereby providing a dependable data-based tool for identifying GBS patients with concomitant cerebrovascular lesions. Risk categorization using probability scores generated by the ANN also facilitated comparison of functional disability trajectories over time. While longitudinal analysis did not identify a statistically significant interaction between temporal factors and risk categories, the stratification method effectively distinguished differing clinical progression patterns among patient subgroups.

## Data Availability

The raw data supporting the conclusions of this article will be made available by the authors, without undue reservation.
