# Supplementary material for: Machine learning-based identification of concomitant stroke and prognostic analysis in patients with Guillain-Barré syndrome: a retrospective study
Source: Front Immunol. 2026 May 7;17:1790415. doi: 10.3389/fimmu.2026.1790415 (PMC13189879; doi:10.3389/fimmu.2026.1790415)
Supplement: Supplementary file 1 [file Table1.docx]

Supplementary Material

**Supplementary Material 1**. Overall missingness profile of variables in the training and internal validation cohorts.

| Variable | Missing Ratio |
| --- | --- |
| Outcome | 0 |
| Sex | 0 |
| Smoking history | 0 |
| Alcohol intake history | 0 |
| History of hypertension | 0 |
| History of diabetes | 0 |
| History of coronary heart disease | 0 |
| History of hyperlipidemia | 0 |
| Type of preceding infection | 0 |
| Age | 0 |
| BMI | 0.180769 |
| Progressive stage | 0 |
| CSF-WBC | 0.042308 |
| CSF-Protein | 0.038462 |
| WBC | 0 |
| NEUT | 0 |
| LYMPH | 0 |
| MONO | 0 |
| NEUT% | 0 |
| LYMPH% | 0 |
| MONO% | 0 |
| Hb | 0 |
| RBC | 0 |
| PLT | 0 |
| Na | 0.026923 |
| K | 0.026923 |
| Cr | 0.019231 |
| eGFR | 0.153846 |
| UA | 0.019231 |
| ALT | 0.015385 |
| AST | 0.015385 |
| HDL-C | 0.134615 |
| LDL-C | 0.134615 |
| CK | 0.103846 |
| CK-MB | 0.096154 |
| PT | 0.042308 |
| INR | 0.042308 |
| APTT | 0.042308 |
| TT | 0.042308 |
| FIB | 0.042308 |
| GLU | 0.134615 |
| HbA1c | 0.165385 |
| FT3 | 0.111538 |
| FT4 | 0.111538 |
| TSH | 0.111538 |
| AISI | 0 |
| LHR | 0.134615 |
| MHR | 0.134615 |

**Supplementary Material 2**. The optimal full set of hyper-parameters of the model.

1. Optimal hyper-parameter set for decision tree: {'ccp_alpha': 0.01, 'max_depth': 3, 'max_features': None, 'min_samples_split': 10}

2. Optimal hyper-parameter set for RF: n_estimators = 300, max_features = 0.3

3. Optimal hyper-parameter set for XGBoost: {'learning_rate': 0.01, 'max_depth': 3, 'n_estimators': 50, 'subsample': 0.8}

4. Optimal hyper-parameter set for LightGBM: {'colsample_bytree': 0.7, 'learning_rate': 0.05, 'max_depth': 5, 'min_child_samples': 40, 'n_estimators': 500, 'num_leaves': 31, 'reg_alpha': 1, 'reg_lambda': 3, 'subsample': 0.7}

5. Optimal hyper-parameter set for SVM: {'C': 1, 'degree': 2, 'gamma': 'scale', 'kernel': 'linear'}

6. Optimal hyper-parameter set for ANN: {'activation': 'relu', 'hidden_layer_sizes': (25,)}

**Supplementary Material 3**. Model performance evaluation in the internal validation cohort.

| Model | AUC | 95% CI Lower | 95% CI Upper | Accuracy | Precision | Sensitivity | Specificity | F1 Score | Kappa | Youden's J | PPV | NPV |
| --- | --- | --- | --- | --- | --- | --- | --- | --- | --- | --- | --- | --- |
| Logistic | 0.815911 | 0.704282 | 0.906755 | 0.743590 | 0.756757 | 0.717949 | 0.769231 | 0.736842 | 0.487179 | 0.487179 | 0.756757 | 0.731707 |
| Decision Tree | 0.710059 | 0.596321 | 0.807186 | 0.717949 | 0.707317 | 0.743590 | 0.692308 | 0.725000 | 0.435897 | 0.435897 | 0.707317 | 0.729730 |
| Random Forest | 0.821170 | 0.716426 | 0.912299 | 0.756410 | 0.763158 | 0.743590 | 0.769231 | 0.753247 | 0.512821 | 0.512821 | 0.763158 | 0.750000 |
| XGBoost | 0.772189 | 0.652520 | 0.872425 | 0.653846 | 0.593750 | 0.974359 | 0.333333 | 0.737864 | 0.307692 | 0.307692 | 0.593750 | 0.928571 |
| LightGBM | 0.777120 | 0.657605 | 0.878372 | 0.756410 | 0.750000 | 0.769231 | 0.743590 | 0.759494 | 0.512821 | 0.512821 | 0.750000 | 0.763158 |
| SVM | 0.827745 | 0.727047 | 0.913648 | 0.743590 | 0.771429 | 0.692308 | 0.794872 | 0.729730 | 0.487179 | 0.487179 | 0.771429 | 0.720930 |
| ANN | 0.838264 | 0.739474 | 0.922883 | 0.782051 | 0.789474 | 0.769231 | 0.794872 | 0.779221 | 0.564103 | 0.564103 | 0.789474 | 0.775000 |


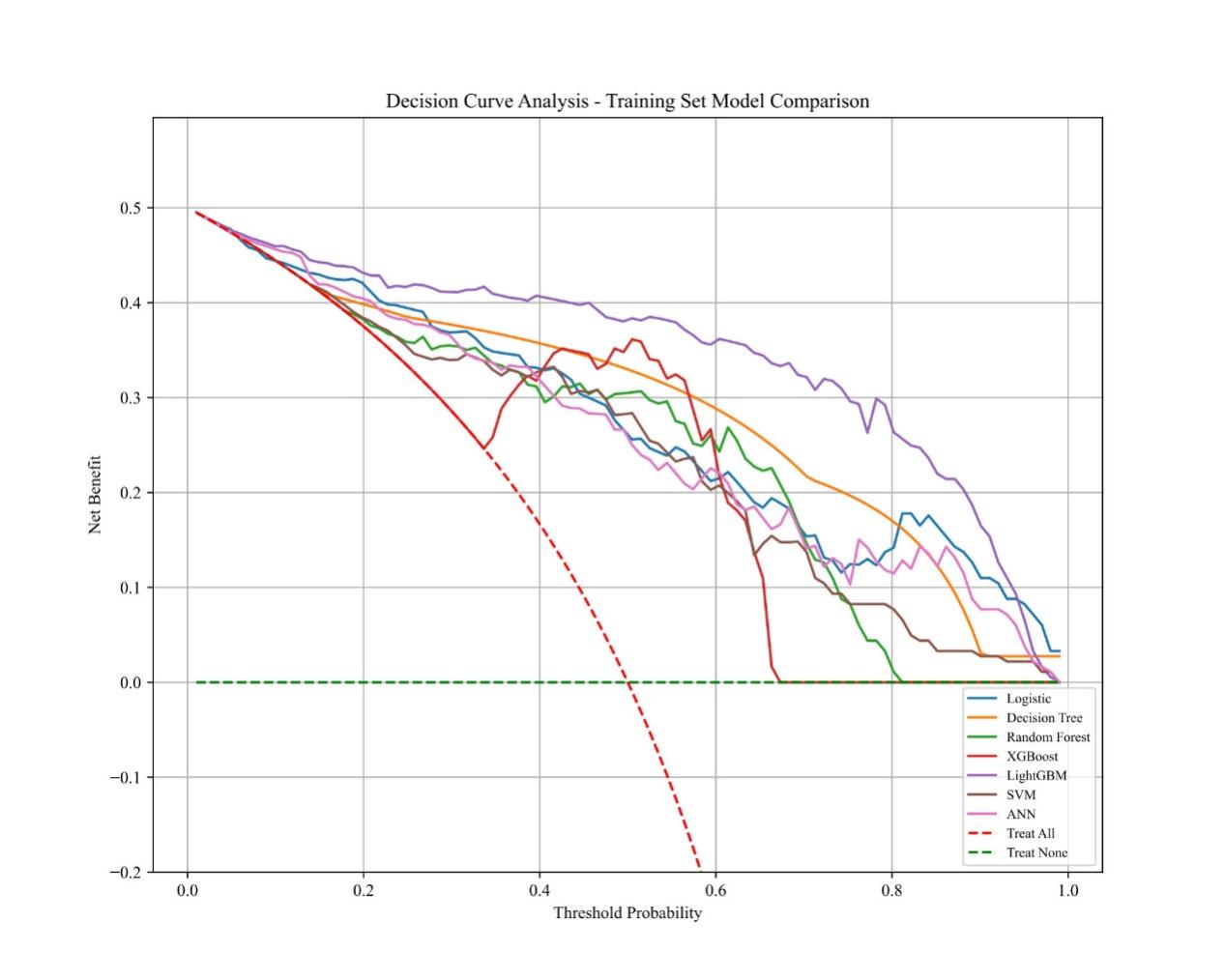

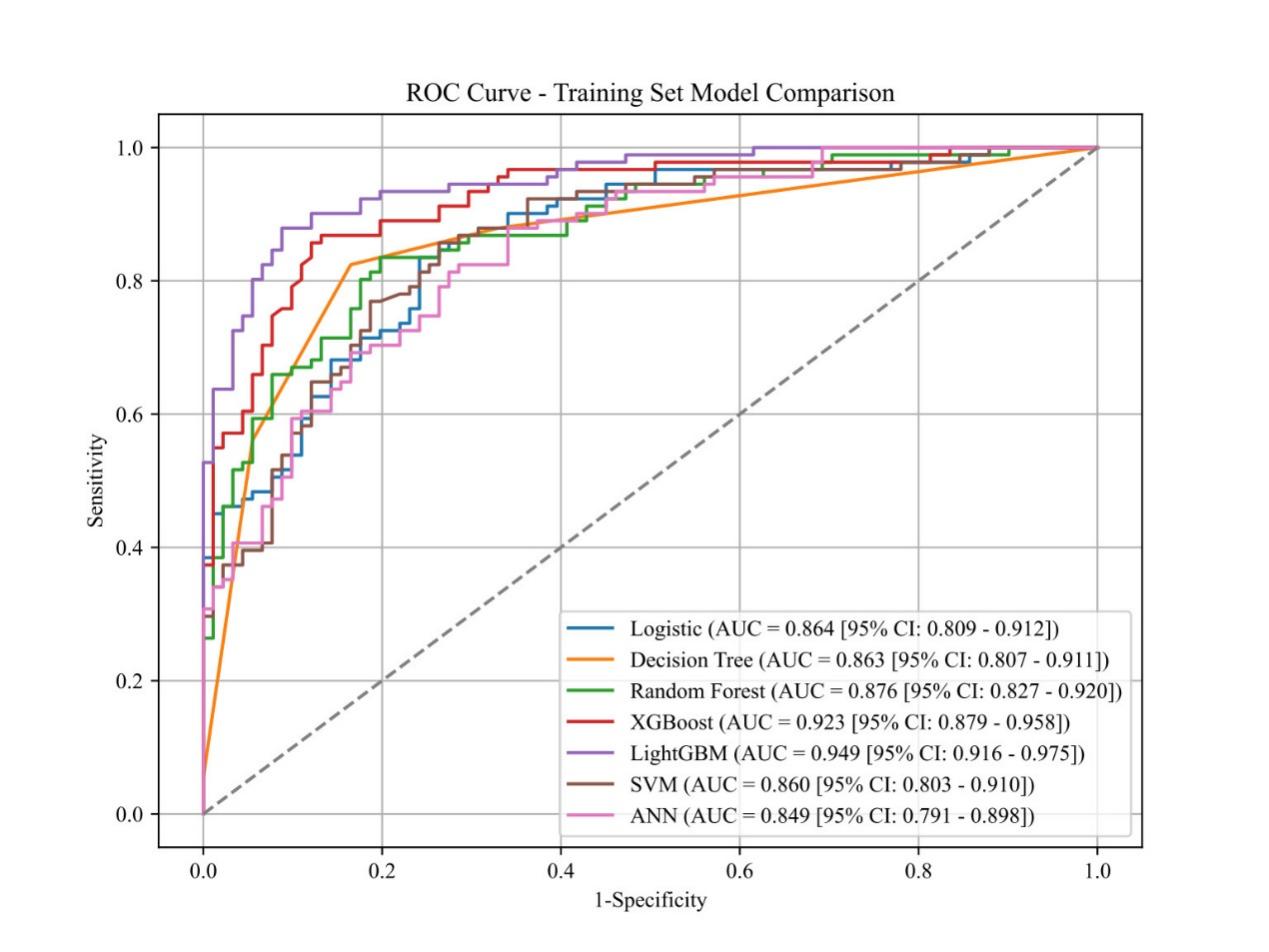
**Supplementary Material 4**. ROC curves, DCA curves, and Calibration curves of the training cohort.


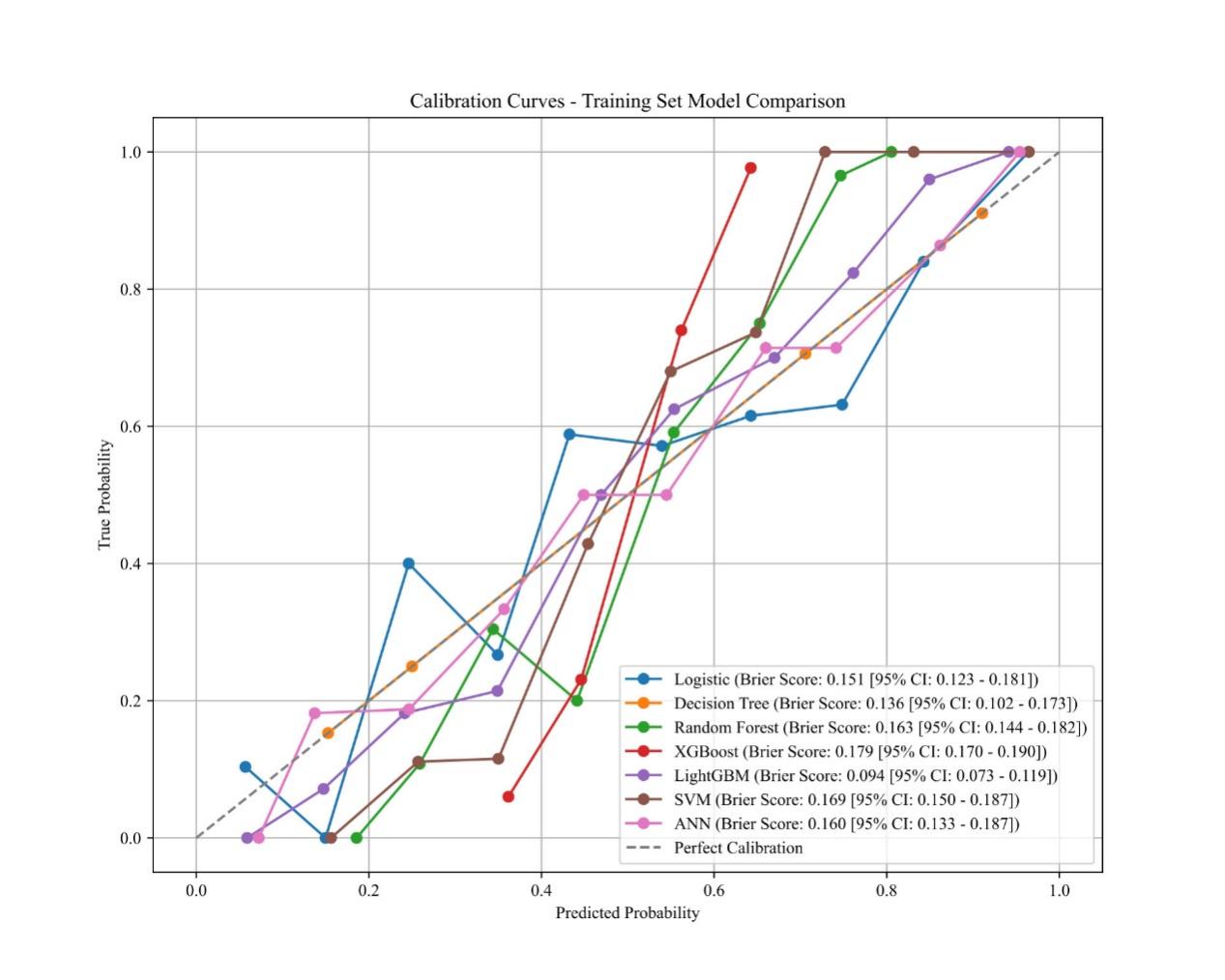


**Supplementary Material 5**. Model performance evaluation in the training cohort.

| Model | AUC | 95% CI Lower | 95% CI Upper | Accuracy | Precision | Sensitivity | Specificity | F1 Score | Kappa | Youden's J | PPV | NPV |
| --- | --- | --- | --- | --- | --- | --- | --- | --- | --- | --- | --- | --- |
| Logistic | 0.864147 | 0.808826 | 0.912302 | 0.763736 | 0.76087 | 0.769231 | 0.758242 | 0.765027 | 0.527473 | 0.527473 | 0.760870 | 0.766667 |
| Decision Tree | 0.863181 | 0.806619 | 0.911277 | 0.774725 | 0.727273 | 0.879121 | 0.670330 | 0.796020 | 0.549451 | 0.549451 | 0.727273 | 0.847222 |
| Random Forest | 0.876464 | 0.827263 | 0.919988 | 0.802198 | 0.783505 | 0.835165 | 0.769231 | 0.808511 | 0.604396 | 0.604396 | 0.783505 | 0.823529 |
| XGBoost | 0.922534 | 0.878636 | 0.958379 | 0.741758 | 0.666667 | 0.967033 | 0.516484 | 0.789238 | 0.483516 | 0.483516 | 0.666667 | 0.940000 |
| LightGBM | 0.949040 | 0.916424 | 0.975067 | 0.884615 | 0.87234 | 0.901099 | 0.868132 | 0.886486 | 0.769231 | 0.769231 | 0.872340 | 0.897727 |
| SVM | 0.859800 | 0.803019 | 0.909997 | 0.780220 | 0.78022 | 0.780220 | 0.780220 | 0.780220 | 0.560440 | 0.560440 | 0.780220 | 0.780220 |
| ANN | 0.848690 | 0.791276 | 0.898453 | 0.763736 | 0.75000 | 0.791209 | 0.736264 | 0.770053 | 0.527473 | 0.527473 | 0.750000 | 0.779070 |


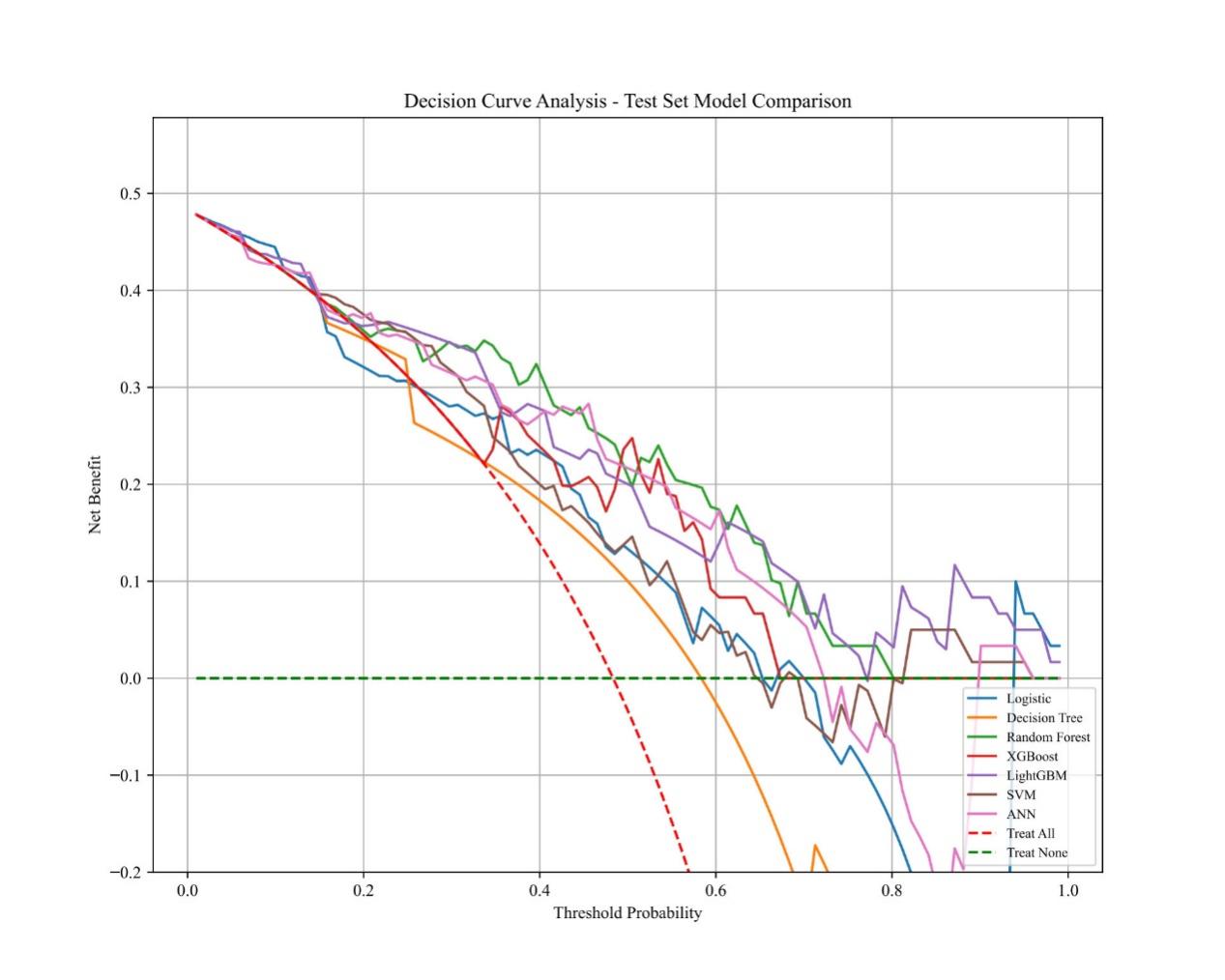

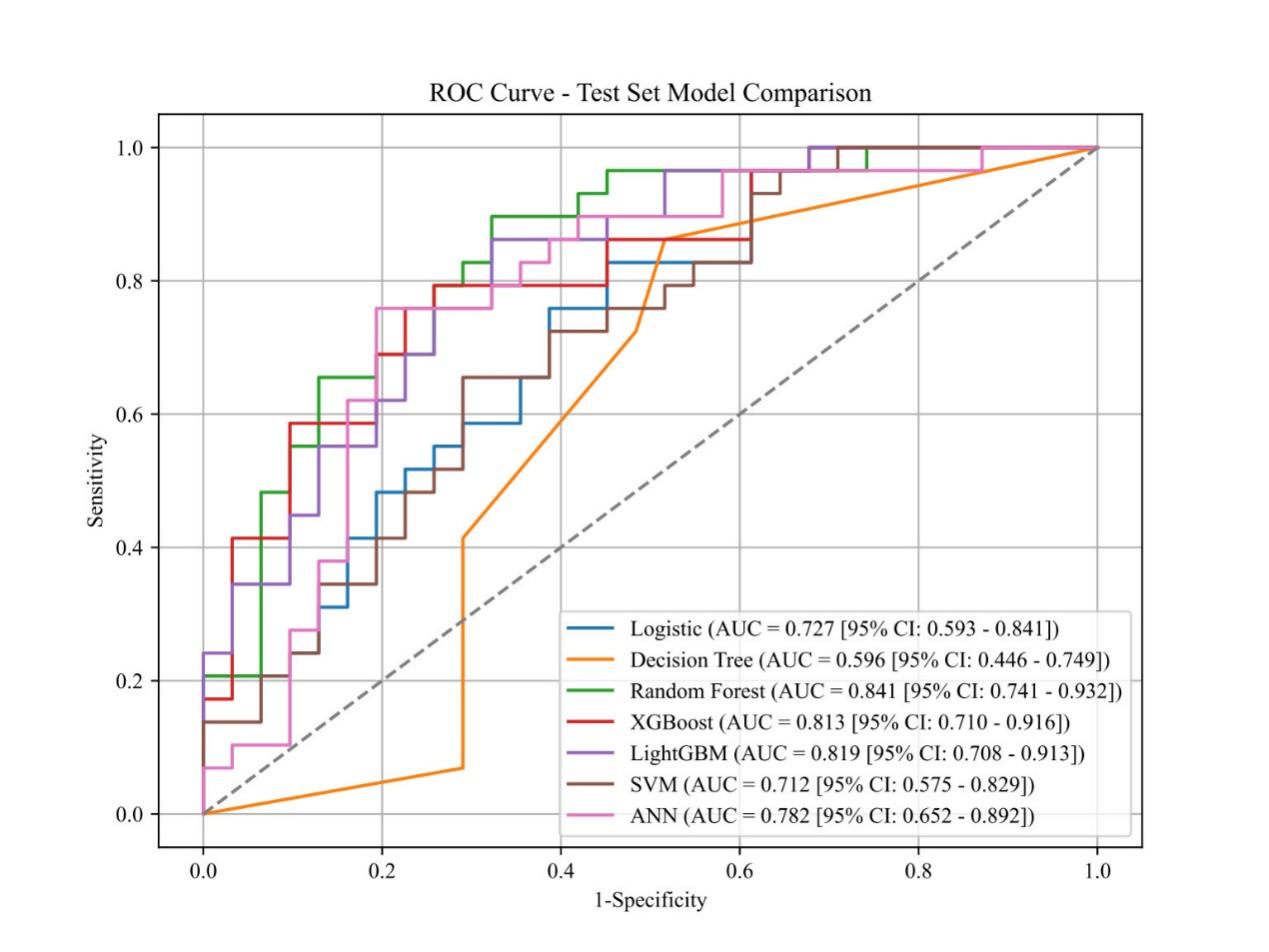
**Supplementary Material 6**. ROC curves, DCA curves, and Calibration curves of the external validation cohort.


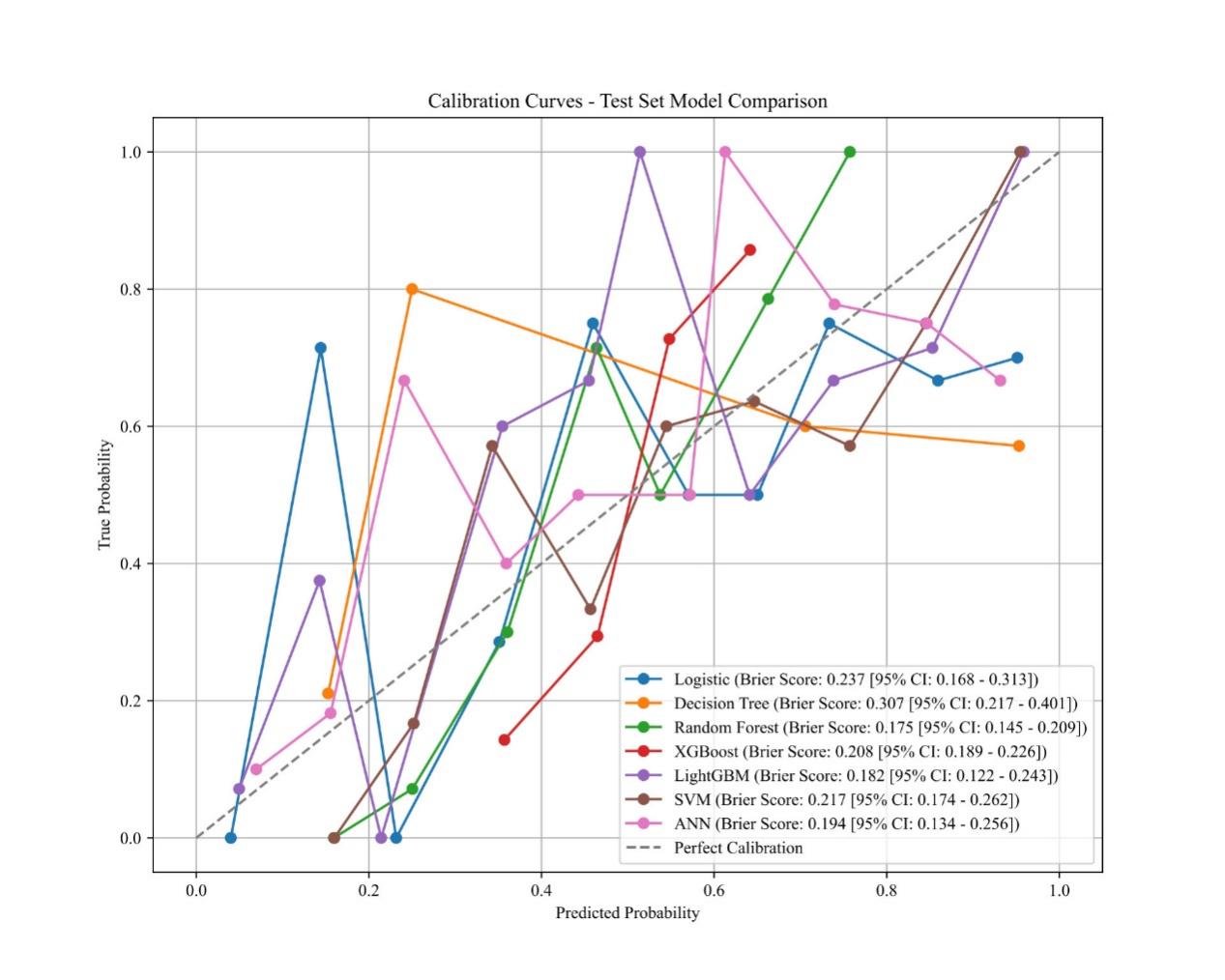


**Supplementary Material 7**. Model performance evaluation in the external validation cohort.

| Model | AUC | 95% CI Lower | 95% CI Upper | Accuracy | Precision | Sensitivity | Specificity | F1 Score | Kappa | Youden's J | PPV | NPV |
| --- | --- | --- | --- | --- | --- | --- | --- | --- | --- | --- | --- | --- |
| Logistic | 0.727475 | 0.592513 | 0.840952 | 0.650000 | 0.633333 | 0.655172 | 0.645161 | 0.644068 | 0.300000 | 0.300334 | 0.633333 | 0.666667 |
| Decision Tree | 0.596218 | 0.445659 | 0.748890 | 0.666667 | 0.609756 | 0.862069 | 0.483871 | 0.714286 | 0.341383 | 0.345940 | 0.609756 | 0.789474 |
| Random Forest | 0.840934 | 0.740768 | 0.932183 | 0.716667 | 0.714286 | 0.689655 | 0.741935 | 0.701754 | 0.432071 | 0.431591 | 0.714286 | 0.718750 |
| XGBoost | 0.813126 | 0.709689 | 0.915837 | 0.650000 | 0.586957 | 0.931034 | 0.387097 | 0.720000 | 0.312227 | 0.318131 | 0.586957 | 0.857143 |
| LightGBM | 0.818687 | 0.708284 | 0.913144 | 0.750000 | 0.733333 | 0.758621 | 0.741935 | 0.745763 | 0.500000 | 0.500556 | 0.733333 | 0.766667 |
| SVM | 0.711902 | 0.575067 | 0.829428 | 0.650000 | 0.617647 | 0.724138 | 0.580645 | 0.666667 | 0.303097 | 0.304783 | 0.617647 | 0.692308 |
| ANN | 0.781980 | 0.651758 | 0.892251 | 0.733333 | 0.760000 | 0.655172 | 0.806452 | 0.703704 | 0.463687 | 0.461624 | 0.760000 | 0.714286 |
